# The de-ubiquitylating enzymes USP26 and USP37 regulate homologous recombination by counteracting RAP80

**DOI:** 10.1093/nar/gkv613

**Published:** 2015-06-22

**Authors:** Dimitris Typas, Martijn S. Luijsterburg, Wouter W. Wiegant, Michaela Diakatou, Angela Helfricht, Peter E. Thijssen, Bram van de Broek, Leon H. Mullenders, Haico van Attikum

**Affiliations:** 1 Department of Human Genetics, Leiden University Medical Center, Einthovenweg 20, 2333 ZC, Leiden, The Netherlands; 2 Biophysics of Cell Signaling, The Netherlands Cancer Institute, Plesmanlaan 121, 1066 CX, Amsterdam, The Netherlands

## Abstract

The faithful repair of DNA double-strand breaks (DSBs) is essential to safeguard genome stability. DSBs elicit a signaling cascade involving the E3 ubiquitin ligases RNF8/RNF168 and the ubiquitin-dependent assembly of the BRCA1-Abraxas-RAP80-MERIT40 complex. The association of BRCA1 with ubiquitin conjugates through RAP80 is known to be inhibitory to DSB repair by homologous recombination (HR). However, the precise regulation of this mechanism remains poorly understood. Through genetic screens we identified USP26 and USP37 as key de-ubiquitylating enzymes (DUBs) that limit the repressive impact of RNF8/RNF168 on HR. Both DUBs are recruited to DSBs where they actively remove RNF168-induced ubiquitin conjugates. Depletion of USP26 or USP37 disrupts the execution of HR and this effect is alleviated by the simultaneous depletion of RAP80. We demonstrate that USP26 and USP37 prevent excessive spreading of RAP80-BRCA1 from DSBs. On the other hand, we also found that USP26 and USP37 promote the efficient association of BRCA1 with PALB2. This suggests that these DUBs limit the ubiquitin-dependent sequestration of BRCA1 via the BRCA1-Abraxas-RAP80-MERIT40 complex, while promoting complex formation and cooperation of BRCA1 with PALB2-BRCA2-RAD51 during HR. These findings reveal a novel ubiquitin-dependent mechanism that regulates distinct BRCA1-containing complexes for efficient repair of DSBs by HR.

## INTRODUCTION

DNA double-strand breaks (DSBs) pose a considerable threat to the stability of the human genome. Their timely repair is not only essential to safeguard genome stability, but also counteracts tumor development (1). Cells activate robust signaling pathways in response to DSBs that coordinate cell cycle progression, changes in chromatin structure and DNA repair (2,3). Eukaryotic cells primarily utilize homologous recombination (HR) or non-homologous end-joining (NHEJ) to remove DSBs from their genomes.

A key feature of the DNA damage response (DDR) is the rapid assembly of signaling and repair factors in the vicinity of DSBs, by progressively modifying histones and DNA repair enzymes (4,5). An initial phosphorylation-dependent cascade of post-translational modifications in DSB-containing chromatin requires the Ataxia Telangiectasia Mutated (ATM) kinase and culminates into the association of MDC1 with phosphorylated histone H2A variant H2AX (γH2AX) (6). The binding of the RNF8 E3 ubiquitin ligase to MDC1 subsequently initiates a ubiquitylation-dependent cascade, involving the recruitment of the E3 ubiquitin ligase RNF168 in cooperation with the E2 ubiquitin-conjugating enzyme UBC13 (7,8). The activity of these enzymes contributes to the ubiquitylation of K13/15 on histone H2A/H2AX (9,10), as well as the ubiquitin-dependent assembly of 53BP1 (11), RAD18 (12) and the BRCA1-Abraxas-RAP80-MERIT40 (or BRCA1-A) complex (13–16) onto DSB-neighboring chromatin.

The RNF8/RNF168-induced ubiquitylation cascade is tightly controlled by sophisticated mechanisms that entail chromatin remodeling enzymes (17–19) and additional ubiquitin ligases (20). Furthermore, it has recently become clear that the removal of ubiquitin by specific de-ubiquitylating enzymes (DUBs) represents an equally important regulatory mechanism in the DDR (21–25). The human genome contains ∼90 potential DUBs that belong to five distinct subfamilies: ubiquitin-specific proteases (USPs), ubiquitin carboxy-terminal hydrolases (UCHs), ovarian tumor proteases (OTUs), Machado-Joseph disease enzymes (MJDs) and JAB1/MPN/MOV34 metalloenzymes (JAMMs). A number of DUBs have been linked to reversing RNF8/RNF168-mediated chromatin ubiquitylation during DNA damage signaling (21,26) and a recent genetic screening approach identified many DUBs with potential roles in the DDR (24).

Although the principles underlying the RNF8 signaling pathway are by now well understood, we are only beginning to comprehend how this pathway is linked to the actual repair of DSBs through the major repair pathways NHEJ (27) and HR (28–30). During HR, the ends of a DSB are resected to expose 3′ single-stranded DNA (ssDNA) overhangs, which are rapidly coated with the ssDNA-binding protein RPA. Following resection, the PALB2 protein is recruited by BRCA1 and subsequently facilitates the assembly of BRCA2 (31,32). This, in turn, promotes the exchange of RPA with RAD51, which drives the search for and pairing with a homologous sequence, as well as the exchange of homologous DNA during the final steps of HR (31–33). BRCA1 is incorporated into distinct multi-protein complexes, including BRCA1-PALB2-BRCA2-RAD51 (BRCC complex) and BRCA1-A (34). Strikingly, while the BRCC complex promotes HR, the BRCA1-A complex functionally antagonizes this repair process by either inhibiting DNA end-resection or sequestering BRCA1 away from HR sites by binding to RNF8/RNF168-ubiquitylated chromatin (16,35–40). These findings suggest that distinct BRCA1-containing complexes can differentially affect HR in a manner dependent on DNA damage-induced ubiquitylation. Remarkably, little is known about the involvement of DUBs in regulating BRCA1-dependent HR.

Through genetic screens we identified the de-ubiquitylating enzymes USP26 and USP37 as key factors whose activities are critical for DSB repair by HR. Mechanistically, we show that by removing RNF168-induced ubiquitin conjugates distal from DSBs, these enzymes prevent the ubiquitin-dependent sequestration of BRCA1 through the BRCA1-A complex, while promoting the association and cooperation of BRCA1 with the BRCC complex during HR. Thus, these enzymes promote HR by limiting the repressive impact of the BRCA1-A complex on this process, as well as by stimulating BRCC complex formation. These findings reveal a novel ubiquitin-dependent mechanism that regulates the functioning of distinct HR complexes at DSBs.

## MATERIALS AND METHODS

### Cell culture

U2OS, HEK293 and VH10-SV40-immortalized cells were grown in Dulbecco's modified Eagle's medium (Gibco) containing 10% Fetal Calf Serum (FCS) (Bodinco BVF). U2OS cells stably expressing FLAG-RNF8 with inducible shRNA against endogenous RNF8, U2OS 2-6-3 cells containing 200 copies of a LacO-containing cassette (∼4 Mbp) and U2OS 2-6-3 cells stably expressing an inducible version of FokI-mCherry-LacR fused to the estrogen receptor and harboring a destabilization domain were previously described (7,15,41–42). The ViraPower system (Life Science) was used to produce lentivirus with mAG- or mCherry-geminin expression vectors (43). U2OS cells stably expressing mAG- or mCherry-geminin were made by standard lentiviral transduction followed by FACS sorting in order to select fluorescent cells.

### Plasmids

A collection of cDNAs encoding FLAG-tagged DUBs, originally generated in Wade Harper's laboratory (44), was obtained from Addgene. An IRES-Puro cassette was amplified by polymerase chain reaction (PCR) and inserted as an EcoRV–EcoRV fragment into the HpaI site of EGFP-C1 (Addgene). The USP26 and USP37 cDNAs were inserted in EGFP-C1-IRES-Puro. Overlap PCR was used to introduce the inactivation mutations C304S into GFP-USP26 and C350S into GFP-USP37. Wild-type and catalytic inactive versions of USP26 and USP37 were also inserted into mCherry-C1. Additional plasmids used are listed in the Supplementary Table.

### Transfections and RNAi interference

siRNA and plasmid transfections were performed using Lipofectamine RNAiMAX (Invitrogen), Lipofectamine 2000 (Invitrogen) and JetPEI (Polyplus Transfection) according to the manufacturer's instructions. Cells were transfected twice with siRNAs (40 nM) within 24 h and examined further 48 h after the second transfection, unless stated otherwise. siRNA sequences are listed in the Supplementary Table.

### Generation of DSBs

Ionizing radiation (IR) was delivered by a YXlon X-ray generator (YXlon International, 200 KV, 10 mA, dose rate 2 Gy/min.). PARP inhibitor (KU-0058948) was used at a concentration of 10 µM.

### Cell survival assay

VH10-SV40 or U2OS cells were transfected with siRNAs, trypsinized, seeded at low density and exposed to IR. Seven days later cells were washed with 0.9% NaCl and stained with methylene blue. Colonies of more than 10 cells were scored.

### FokI assays

U2OS 2-6-3 cells expressing inducible FokI-mCherry-LacR (42) were treated with 300 nM 4-OHT and 1 μM Shield-I for 5 h. Subsequently, cells were fixed with formaldehyde and immunostained with the indicated antibodies.

### UV-A laser micro-irradiation

U2OS cells were grown on 18 mm coverslips and sensitized with 10 μM 5′-bromo-2-deoxyuridine (BrdU) for 24 h as described (18,45). For micro-irradiation, the cells were placed in a Chamlide TC-A live-cell imaging chamber that was mounted on the stage of a Leica DM IRBE widefield microscope stand (Leica, Wetzlar, Germany) integrated with a pulsed nitrogen laser (Micropoint Ablation Laser System; Photonic Instruments, Inc., Belfast, Ireland). The pulsed nitrogen laser (16 Hz, 364 nm) was directly coupled to the epifluorescence path of the microscope and focused through a Leica 40× HCX PLAN APO 1.25–0.75 oil-immersion objective. The growth medium was replaced by CO_2_-independent Leibovitz's L15 medium supplemented with 10% FCS and penicillin-streptomycin and cells were kept at 37°C. The laser output power was set to 78 to generate strictly localized sub-nuclear DNA damage. Following micro-irradiation, cells were incubated for the indicated time-points at 37°C in Leibovitz's L15 and subsequently fixed with 4% formaldehyde before immunostaining. Typically, an average of 50 cells was micro-irradiated (two iterations per pixel) within 10–15 min using Andor IQ software.

### Multiphoton laser micro-irradiation

U2OS cells were grown on 18 mm coverslips. Subsequently, cells were placed in a Chamlide CMB magnetic chamber and the growth medium was replaced by CO_2_-independent Leibovitz's L15 medium supplemented with 10% FCS and penicillin-streptomycin. Laser micro-irradiation was carried out on a Leica SP5 confocal microscope equipped with an environmental chamber set to 37°C. DSB-containing tracks (1.5 μm width) were generated with a Mira mode-locked titanium-sapphire (Ti:Sapphire) laser (*λ* = 800 nm, pulse length = 200 fs, repetition rate = 76 MHz, output power = 80 mW) using a UV-transmitting 63× 1.4 NA oil immersion objective (HCX PL APO; Leica). Confocal images were recorded before and after laser irradiation at 5 or 10 s time intervals over a period of 5–10 min.

### Microscopy analysis

Images of fixed cells were acquired on a Zeiss AxioImager D2 widefield fluorescence microscope equipped with 40, 63 and 100× PLAN APO (1.4 NA) oil-immersion objectives (Zeiss) and an HXP 120 metal-halide lamp used for excitation. Fluorescent probes were detected using previously described filters (46). Images were recorded using ZEN 2012 software and analyzed using ImageJ. Alternatively, images were acquired on a Leica SP5 confocal microscope equipped with a 63× 1.4 NA oil immersion objective (HCX PL APO; Leica). Images were recorded using LAS AF software and analyzed using Image J. The average reflects the quantification of 50–150 cells from three independent experiments.

### IRIF analysis

PALB2, RAD51, RAP80 and BRCA1 (Supplementary Figure S2B) IRIF were analyzed in U2OS cells 6 h after 10 Gy, RPA and CtIP IRIF were assayed 4 h after 10 Gy, whereas conjugated ubiquitin (FK2), γH2AX, MDC1, FLAG-RNF8, RNF168, 53BP1, RAP80 and BRCA1 (Supplementary Figure S2B) IRIF were examined 1 h after 2 Gy, unless stated otherwise. IRIF were evaluated in ImageJ, using a custom-built macro that enabled automatic and objective analysis of the foci. Full details of this macro will be published elsewhere. In brief, cell nuclei were detected by thresholding the (median-filtered) DAPI signal, after which touching nuclei were separated by a watershed operation. The foci signal was background-subtracted using a Difference of Gaussians filter. For every nucleus, foci were identified as regions of adjacent pixels satisfying the following criteria: (i) the gray value exceeds the nuclear background signal by a set number of times (typically 2–4×) the median background standard deviation of all nuclei in the image, and is higher than a user-defined absolute minimum value; (ii) the area is larger than a defined area (typically two pixels). These parameters were optimized for every experiment by manually comparing the detected foci with the original signal.

### Immunofluorescent labeling

Immunofluoresecent labeling was carried out as described previously (18,19). Briefly, cells were grown on glass coverslips and treated as indicated in the figure legends. Subsequently, cells were washed with phosphate buffered saline (PBS), then fixed with 2% formaldehyde for 20 min and permeabilized with 0.25% Triton X-100 in PBS for 5 min. Cells were rinsed with PBS and then treated with 100 mM glycine in PBS for 10 min to block unreacted aldehyde groups. Finally, cells were equilibrated in PBS containing 0.5% BSA and 0.05% Tween 20, and incubated with primary antibodies. Detection was done using goat anti-mouse or goat anti-rabbit IgG coupled to Alexa 488, 555 or 647 (Invitrogen Molecular Probes). Samples were incubated with 0.1 μg/ml DAPI and mounted in Polymount. Primary antibodies and secondary antibodies are listed in the Supplementary Table.

### Western blotting

Cell extracts were generated by boiling cell pellets in Laemmli buffer, separated by sodium dodecyl sulphate-polyacrylamide gel electrophoresis and transferred to PVDF membranes (Millipore). Membranes were probed with the antibodies listed in the Supplementary Table followed by protein detection using the Odyssey infrared imaging scanning system (LI-COR Biosciences).

### HR and NHEJ assay

HEK293 and U2OS cells containing a stably integrated copy of either the DR-GFP of EJ5-GFP reporter were used to measure the repair of I-SceI-induced DSBs by HR and NHEJ, respectively (47,48). Briefly, 48 h after siRNA transfection, cells were transfected with the I-SceI expression vector pCBASce and an mCherry expression vector (47). About 48 or 72 h later the fraction of GFP-positive cells among the mCherry-positive cells was determined by FACS on a LSRII flow cytometer (BD Bioscience) using FACSDiva software version 5.0.3. Quantifications were performed using Flowing Software (www.flowingsoftware.com).

### Cell cycle profiling

For cell cycle analysis cells were fixed in 70% ethanol, followed by DNA staining with 50 μg/ml propidium iodide in the presence of RNase A (0.1 mg/ml). Cell sorting was performed on a LSRII flow cytometer (BD Bioscience) using FACSDiva software (version 5.0.3; BD). Quantifications were performed using Flowing Software.

### RT-qPCR-based gene expression analysis

RNA isolation, reverse transcription (RT)-based cDNA synthesis and quantitative (q)PCR were carried out as previously described (46). The primers used are listed in the Supplementary Table.

### Immunoprecipitation (IP) of endogenous PALB2 and RAP80

Cell were washed with PBS, trypsinized and collected by centrifugation. Cell-pellets were lysed for 1 h under rotation in 1 ml of EBC-150 buffer (50 mM Tris pH 7.3, 150 mM NaCl, 0.5% NP-40, 1 mM MgCl_2_, protease inhibitors) complemented with 500U of benzonase. Subsequently, lysates were centrifuged at full speed in an Eppendorf microcentrifuge and 50 μl fractions were collected as input. The remaining lysate fractions were placed into new tubes containing 2 μg of the indicated antibodies and rotated for 3 h at 4°C. Then, 20 μl of protein A beads were added to the suspension and samples were rotated for 1 h at 4°C. The beads were washed six times with 1 ml of EBC-EDTA buffer (50 mM Tris pH 7.3, 150 mM NaCl, 0.5% NP-40, 1 mM EDTA, protease inhibitors), after which 20 μl of 2× Laemmli buffer was added to the beads as well as to 20 μl of input material. Samples were boiled at 95°C for 15 min and loaded on a polyacrylamide gel.

### Denaturing GFP-H2A immunoprecipitation (IP)

Cells were lysed in 1 volume of lysis buffer (20 mM Tris pH 7.5, 50 mM NaCl, 0.5% NP-40, 1% sodium deoxycholate, 1% sodium dodecyl sulphate (SDS), 2.5mM MgCl_2_). Cell lysates were adjusted by adding four volumes of wash buffer (20 mM Tris pH 7.5, 50 mM NaCl, 0.5% NP-40, 0.5% sodium deoxycholate, 0.5% SDS). Subsequently, benzonase was added to a final concentration of 0.25 U/μl to degrade nucleosomal DNA and samples were incubated for 1 h at room temperature. After centrifuging at 15 000 *g* for 5 min, supernatants were collected and transferred to equilibrated GFP-Trap^®^_A beads. Cell lysates were incubated on the GFP-Trap^®^_A beads under constant mixing for 2 h at room temperature. Beads were collected and washed six times with wash buffer. After the final wash, beads were collected, boiled in SDS sample buffer and samples were loaded on a polyacrylamide gel. All buffers were supplemented with protease cocktail inhibitor.

## RESULTS

### A screen for DUBs reveals novel regulators of 53BP1 and RAD51

**Figure 1.**
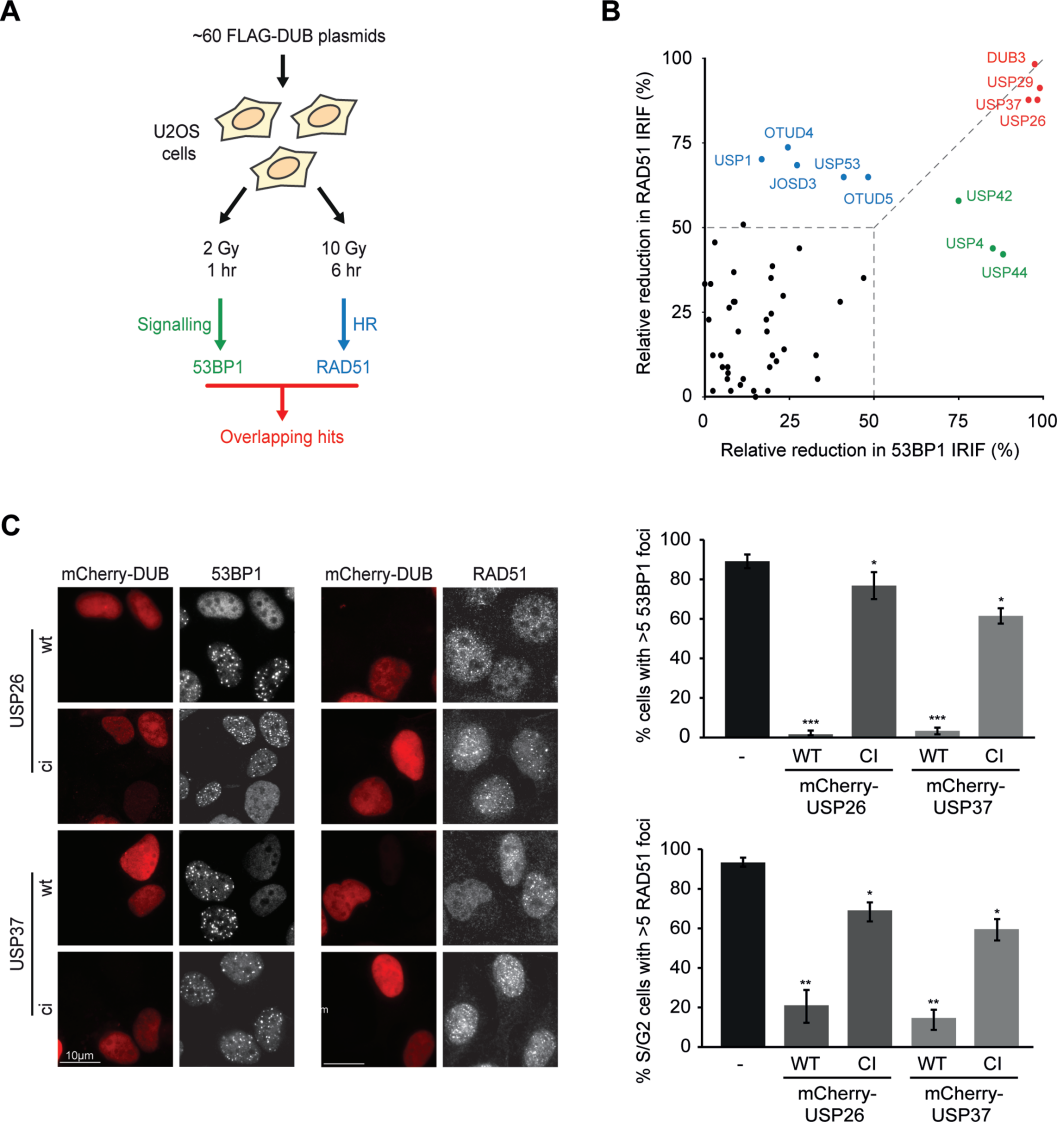
DUB screen for regulators of 53BP1 and RAD51 IRIF formation. (**A**) Experimental design of the DUB over-expression screens. (**B**) Bi-dimensional representation of the relative decrease in 53BP1 (x-axis) and RAD51 (y-axis) IRIF formation upon over-expression of FLAG-tagged DUBs. (**C**) Impact of the expression of wild-type (WT) or catalytic inactive (CI) mCherry-tagged DUBs (red) on 53BP1 (white; left panel) or RAD51 IRIF formation (white; right panel) in mAG-geminin-expressing (images not shown) S/G2 cells. Quantified data are represented as mean ± S.D. (*n* = 3). ns = non-significant, *P < 0.05, **P < 0.01, ***P < 0.001 (student's *t*-test). See also Supplementary Figure S1.

The BRCA1 protein is incorporated into distinct multi-protein complexes that are not all competent in promoting HR. While the BRCA1-PALB2-BRCA2-RAD51 (BRCC) complex promotes HR, the BRCA1-Abraxas-RAP80-MERIT40 (BRCA1-A) complex functionally antagonizes this repair process either by limiting DNA end-resection or sequestering BRCA1 away from HR sites by binding to RNF8/RNF168-ubiquitylated chromatin (16,35–40). These findings suggest that distinct BRCA1-containing complexes can differentially affect HR in a manner that depends on the activity of the RNF8/RNF168 ubiquitin response. Although the responsible E3 ubiquitin ligases RNF8 and RNF168 have been characterized (7–8,13–15), potential DUBs that play a role in this ubiquitin-dependent regulation of HR remain elusive. In order to identify such proteins, we performed an over-expression screen using a FLAG-tagged cDNA library of ∼60 human DUBs (Supplementary Figure S1A) in human U2OS cells (Figure 1A). Specifically, we monitored if DUB over-expression simultaneously antagonizes the IR-induced formation of 53BP1 foci, a read-out for RNF168-mediated ubiquitylation (11) as well as the IR-induced focal accumulation of RAD51, a measure of HR efficiency. Given that 53BP1 directly binds to RNF168-induced ubiquitin conjugates (9,11), we reasoned that DUBs modulating both these processes are likely to regulate RNF168-mediated HR events.

Imaging-based analysis revealed that most DUBs did not appreciably alter the IR-induced accumulation of 53BP1 or RAD51 (Figure 1B; black circles—see also Supplementary Figure S1B and C). However, a subset of DUBs predominantly impinged on 53BP1 accrual (Figure 1B; green circles), such as the earlier reported enzyme USP44, (21) (Supplementary Figure S1D), while another set of DUBs mainly impacted RAD51 foci formation (Figure 1B; blue circles), including the previously described USP1 (49). Only a small number of DUBs strongly affected both 53BP1 and RAD51 recruitment simultaneously (Figure 1B; red circles; >85% reduction in foci formation), including USP29, an enzyme linked to H2A de-ubiquitylation (21) and the recently reported HR modulator DUB3 (Supplementary Figure S1D and E) (50). The fact that we identified various published DUBs demonstrates the validity of our screen. Importantly, among the DUBs that suppressed both 53BP1 and RAD51 IRIF formation, USP26 and USP37 emerged as novel candidates (Figure 1B and Supplementary Figure S1D and E). Counteracting 53BP1 or RAD51 IRIF formation is not a general feature of the USP family, since many USP enzymes did not affect this process (Supplementary Figure S1B–E; see USP49 as a representative example). This appears to be also true for the other DUB families, as we could not distinguish a common pattern in the impact of DUBs on 53BP1 or RAD51 foci formation within UCH, MJD, JAMM, OTU or unclassified DUBs (Supplementary Figure S1B–E), suggesting this is a unique property of the identified enzymes. Thus, our screen identified USP26 and USP37 as potential novel regulators of 53BP1 and RAD51.

### USP26 and USP37 reverse RNF168-induced ubiquitylation at DSBs

**Figure 2.**
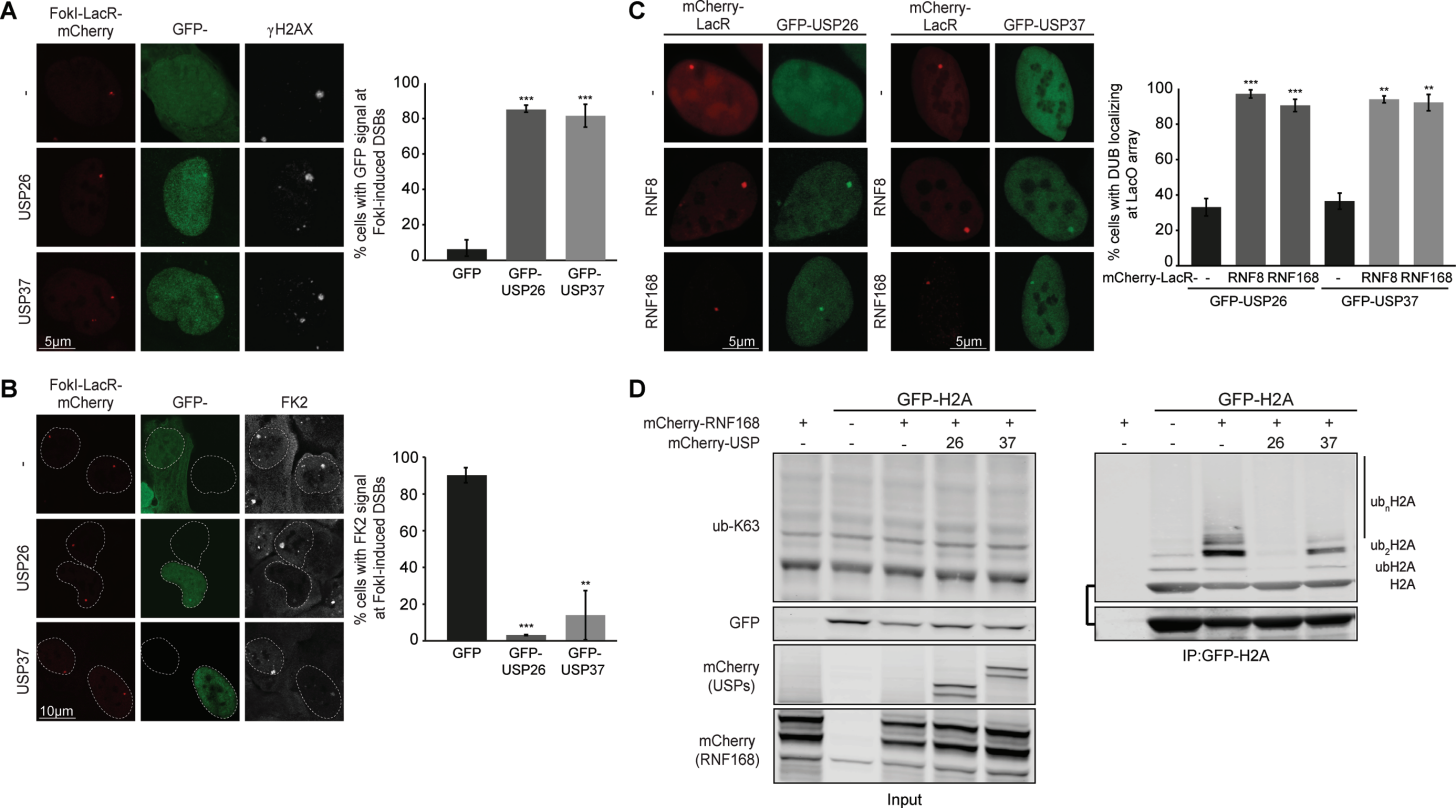
USP26 and USP37 accumulate at DSBs and regulate chromatin ubiquitylation. (**A**) Recruitment of the indicated GFP fusion proteins to FokI-mCherry-LacR-induced DSBs marked by γH2AX (white) in cells containing a LacO array. (**B**) As in A, but stained for conjugated ubiquitin (FK2; white). (**C**) Recruitment of GFP-tagged DUBs upon tethering of the indicated mCherry-LacR fusion proteins in cells containing a LacO array. (**D**) IP of GFP-H2A under denaturing conditions in the absence or presence of mCherry-RNF168 and the indicated mCherry-tagged DUBs. Blots were probed for K63-linked ubiquitin chains. ub_n_H2A, ub_2_H2A and ubH2A indicate poly-ubiquitylated, di-ubiquitylated and mono-ubiquitylated forms of GFP-H2A, respectively. All images were obtained using a confocal microscope. ns = non-significant, *P < 0.05, **P < 0.01, ***P < 0.001 (student's *t*-test). Quantified data are represented as mean ± S.D. (*n* = 3).

To validate and extend these findings, we generated mCherry- and GFP-tagged versions of USP26 and USP37. Over-expression of these DUBs did not alter the accumulation of γH2AX, MDC1 and FLAG-RNF8 (Supplementary Figure S2A), yet abrogated all events downstream of ubiquitin conjugation, including the accrual of ubiquitin-binding factors RNF168, RAP80, BRCA1 and 53BP1 after IR in a catalytic-dependent manner (Figure 1C and Supplementary Figure S2B). Both DUBs were rapidly recruited to laser-induced DNA damage tracks (Supplementary Figure S2C). Given the multitude of DNA lesions inflicted by laser micro-irradiation, we additionally used a U2OS cell line in which DSBs are specifically induced by targeting the LacR-tagged FokI nuclease to a genomic locus containing LacO repeats (42). In line with the results obtained by laser micro-irradiation, both USP26 and USP37 accumulated at FokI-induced DSBs marked by γH2AX (Figure 2A and 2B). Importantly, USP26 and USP37 were able to remove ubiquitin conjugates at these DSBs in a manner dependent on their catalytic activity (Figure 2B and Supplementary Figure S2B (FK2 foci)).

These findings suggest that both DUBs can directly reverse RNF168-induced ubiquitin conjugation. Indeed, when mCherry-LacR-RNF8 or mCherry-LacR-RNF168 was tethered to a LacO array to induce local chromatin ubiquitylation and 53BP1 recruitment (18,45,51), robust accumulation of both DUBs was observed (Figure 2C), indicating that these DUBs can recognize RNF8/RNF168-induced ubiquitin moieties. However, when these DUBs were over-expressed in cells with LacR-RNF168 tethered to the array, we could detect a near complete removal of RNF168-induced ubiquitylation and loss of 53BP1 accumulation (Supplementary Figure S2D and E), suggesting that these DUBs can specifically remove RNF168-cataylzyed ubiquitin chains that are bound by 53BP1. RNF168 targets H2A-type histones for ubiquitylation (9,10). Interestingly, ectopic expression of USP37 moderately decreased RNF168-induced H2A ubiquitylation (9,52), while expression of USP26 nearly eliminated such ubiquitylation (Figure 2D). Importantly, K48-, K63- and total-ubiquitylation (FK2) levels, as well as mono-ubiquitylation of H2B were not altered upon moderate over-expression of either DUB, indicating that these enzymes do not act non-specifically on ubiquitylated proteins (Supplementary Figure S2F). Thus, our results suggest that USP26 and USP37 are able to bind to chromatin modified by RNF8/RNF168 and reverse ubiquitylation induced by these E3 ligases at DSBs.

### Loss of USP26 or USP37 impairs DSB repair

**Figure 3.**
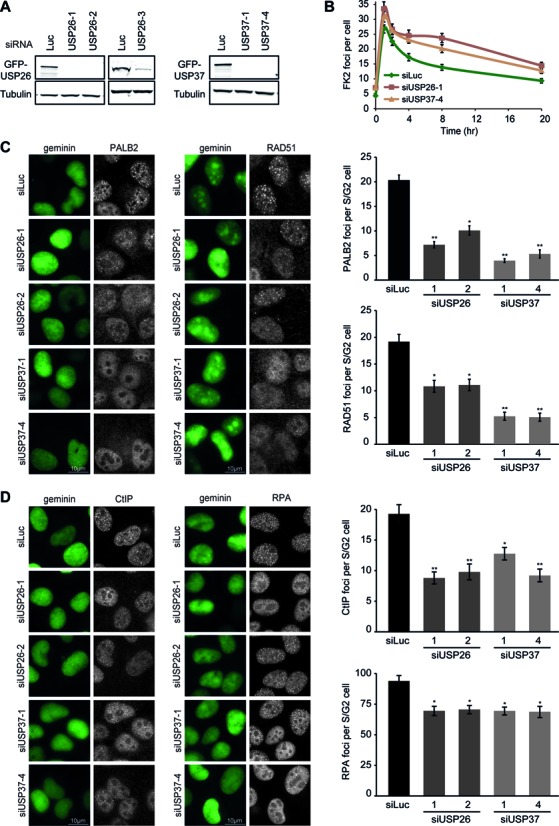
USP26 or USP37 depletion abrogates the formation of PALB2–RAD51 complex at DSBs. (**A**) Western blot analysis of GFP-USP26 or GFP-USP37 expression in cells treated with the indicated siRNAs. (**B**) Effect of DUB depletion on conjugated ubiquitin (FK2) IRIF formation in time. Note that 0 h indicates non-irradiated cells. (**C**) Effect of DUB depletion on RAD51 (white; left panel) or PALB2 (white; right panel) IRIF formation in mAG-geminin-expressing (green) S/G2 cells. (**D**) Effect of DUB depletion on CtIP (white; left panel) or RPA (white; right panel) IRIF formation in mAG-geminin-expressing (green) S/G2 cells. ns = non-significant, *P < 0.05, **P < 0.01, ***P < 0.001 (student's *t*-test). Quantified data are represented as mean ± S.E.M (*n* = 3).

To address if the identified DUBs play a role in HR under physiological conditions, USP26 and USP37 were depleted using independent siRNAs. Immunoblotting (Figure 3A and Supplementary Figure S3B) and RT-qPCR (Supplementary Figure S3A and B) analysis confirmed that the protein and mRNA levels of both DUBs were dramatically reduced. Loss of these DUBs led to a significant increase in IR-induced ubiquitin conjugates (Figure 3B) and accumulation of the ubiquitin-binding protein 53BP1 (Supplementary Figure S3C), suggesting that USP26 and USP37 control the levels of DSB-induced ubiquitylation. The combined knockdown of USP26 and USP37 did not have an additive effect on the rate of 53BP1 IRIF removal, suggesting that these DUBs have non-redundant roles in the DDR (Supplementary Figure S3D).

In order to specifically monitor S/G2 cells, in which HR can take place, we generated U2OS cells stably expressing a monomeric Azami-Green (mAG)-tagged variant of the S/G2 marker geminin. The expression patterns of mAG-geminin correlated with progression through the cell cycle, showing that this cell line is a valuable tool to identify S/G2 cells (Supplementary Figure S3E). When specifically analyzing mAG-positive cells, we found to our surprise that depletion of USP26 or USP37 resulted in a clear reduction in IR-induced PALB2 and RAD51 foci (Figure 3C). In further agreement with a physiological role of USP26 and USP37 in regulating HR, we found that loss of either DUB impaired the formation of CtIP and RPA foci, which is indicative of reduced DNA-end resection (Figure 3D).

**Figure 4.**
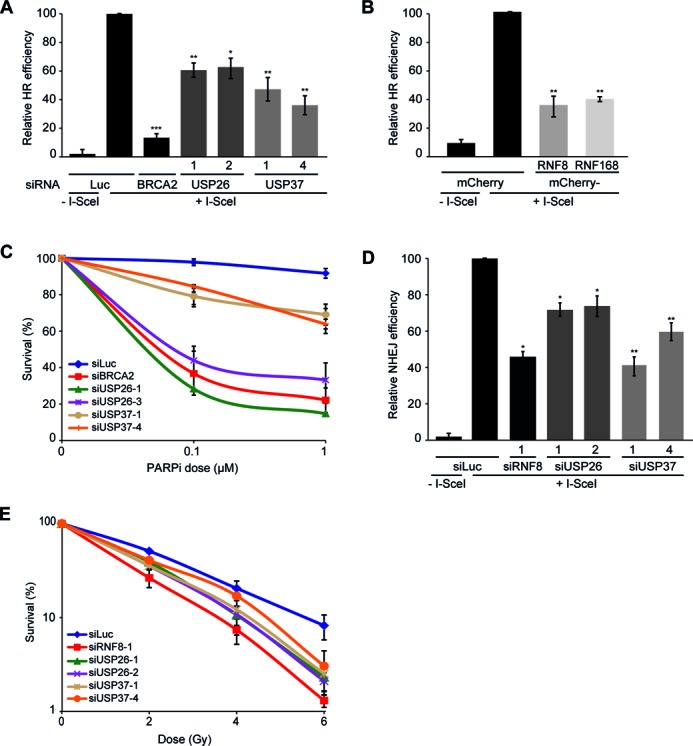
USP26 or USP37 depletion disrupts HR and NHEJ and sensitizes cells to DNA damaging agents. (**A**) Impact of the indicated siRNAs on HR efficiency measured using the DR-GFP reporter. (**B**) Impact of the expression of the indicated mCherry-fusion proteins on HR efficiency using the DR-GFP reporter. (**C**) Clonogenic survival of VH10-SV40 cells that were transfected with the indicated siRNAs and exposed to PARP inhibitor. (**D**) Impact of the indicated siRNAs on NHEJ efficiency measured using the EJ5-GFP reporter. (**E**) Clonogenic survival of IR-exposed U2OS cells transfected with the indicated siRNAs. ns = non-significant, *P < 0.05, **P < 0.01, ***P < 0.001 (student's *t*-test). Quantified data are represented as mean ± S.D. (*n* = 3).

These results suggest a role for USP26 and USP37 in regulating HR. To directly examine this, we employed the DR-GFP reporter to measure DSB repair by HR (47). Flow cytometric analysis of DR-GFP cells confirmed that USP26 or USP37 depletion leads to a significant defect in HR (Figure 4A). Interestingly, no additive effect was apparent upon co-depletion of USP26 and USP37 (Supplementary Figure S3F), suggesting that USP26 and USP37 have non-redundant roles in HR. Over-expression of mCherry-tagged RNF8 or RNF168 also strongly inhibited HR (Figure 4B). Thus, the over-expression of RNF8/168 phenocopied the depletion of USP26 or USP37 as both conditions trigger excessive chromatin ubiquitylation. The effects on HR were not due to alterations in the cell cycle as cell cycle profiles were unchanged under these conditions (Supplementary Figure S4A and B). Depletion of USP26 or USP37 also rendered cells highly sensitive to a poly(ADP-ribose) polymerase (PARP) inhibitor, which is a hallmark of HR-deficient cells such as those lacking BRCA2 (Figure 4C) (53).

Having shown that USP26 and USP37 regulate HR, we next sought to address if these enzymes affect the other major DSB repair pathway, NHEJ. Using the flow cytometry-based EJ5-GFP reporter assay to monitor NHEJ efficiency (48), we found that USP26 or USP37 depletion substantially impaired this repair pathway (Figure 4D). In line with a general defect in DSB repair, the combined knockdown of USP26 and USP37 led to a delay in the clearance of IR-induced γH2AX foci (Supplementary Figure S4C), whereas depletion of either DUB rendered cells sensitive to IR (Figure 4E). Collectively, this work reveals USP26 and USP37 as novel factors that are required for DSB repair. Notably, although we originally identified USP26 and USP37 in an over-expression screen for factors that impair RAD51 foci formation, we find here that the knockdown of these enzymes results in the same phenotype. We next sought to elucidate the mechanism underlying this observation.

### USP26 or USP37 antagonize ubiquitin-dependent spreading of RAP80-BRCA1

**Figure 5.**
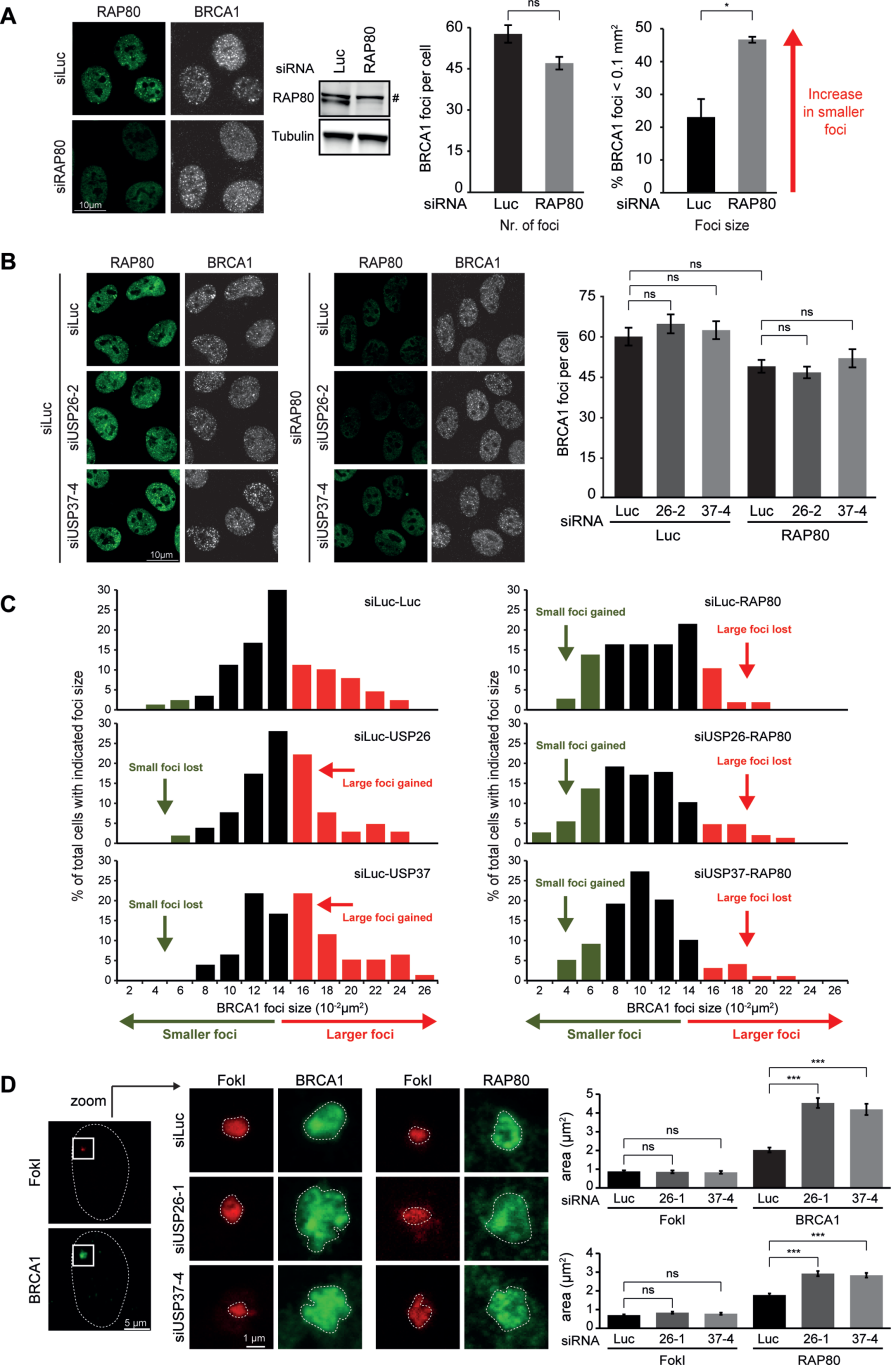
RAP80 depletion restores the formation of small BRCA1 IRIF indicative of HR centers in USP26 and USP37 knockdown cells. (**A**) Effect of RAP80 depletion on RAP80 (green) and BRCA1 (white) IRIF formation (left and middle panel) and BRCA1 IRIF size (right panel). (**B**) Effect of DUB and RAP80 depletion on RAP80 (green) and BRCA1 (white) IRIF formation. (**C**) Histograms of BRCA1 foci size in cells treated with the indicated siRNAs. Green indicates small foci of typical HR size, while red indicates larger foci of the size observed for signaling factors. (**D**) Impact of DUB depletion on the relative position/expansion of the indicated factors (BRCA1 or RAP80; green) upon DSB induction by FokI-mCherry-LacR (red). ns = non-significant, *P < 0.05, **P < 0.01, ***P < 0.001 (student's *t*-test). ^#^denotes a non-specific band. Quantified data are represented as mean ± S.E.M. (*n* = 2).

To gain insight into the mechanism that disrupts HR under conditions of excessive ubiquitylation, we turned our attention to the BRCA1-A complex, which through RAP80 drives the ubiquitin-dependent recruitment of BRCA1 to RNF8/RNF168-modified chromatin (7,13–16,35–37). Recent work unveiled that RAP80-mediated recruitment of BRCA1 counteracts HR (38,39), most likely by inhibiting DNA-end resection or possibly through sequestering BRCA1 from HR sites (38,40). We reasoned that USP26 and USP37 may antagonize the RAP80-dependent sequestration of BRCA1 by removing RNF8/RNF168-mediated ubiquitylation and thereby promote HR. To address this, we established a quantitative, computer-assisted approach to measure BRCA1 foci size. In agreement with an earlier report (38), we found that BRCA1 foci were not reduced in number, but rather were considerably smaller in size following depletion of RAP80 (Figure 5A). In contrast, depletion of either DUB had the opposite effect, leading to an increase of larger BRCA1 foci without affecting the total number of foci (Figure 5B, C and Supplementary Figure S4D). Remarkably, depletion of RAP80 in DUB knockdown cells completely rescued the shift toward larger foci and led to a reappearance of small BRCA1 foci (Figure 5B and C). If USP26 and USP37 counteract RAP80-dependent sequestration of BRCA1, it would be expected that loss of these DUBs would trigger more extensive spreading of the BRCA1-A complex into DSB-flanking chromatin. To test this hypothesis, we induced DSBs by tethering the FokI nuclease to chromatin and subsequently monitored the spreading of ubiquitin and ubiquitin-binding factors by confocal microscopy. In line with our hypothesis, loss of either DUB led to increased spreading not only of RAP80 and BRCA1 (Figure 5D), but also FK2 and 53BP1 (Supplementary Figure S4E) away from the FokI-induced DSBs. This suggests that USP26 and USP37 limit the spreading of chromatin ubiquitylation at DSBs, thereby counteracting sequestration of the BRCA1-A complex.

### Loss of USP26 or USP37 impairs HR by counteracting RAP80-dependent sequestration of BRCA1

**Figure 6.**
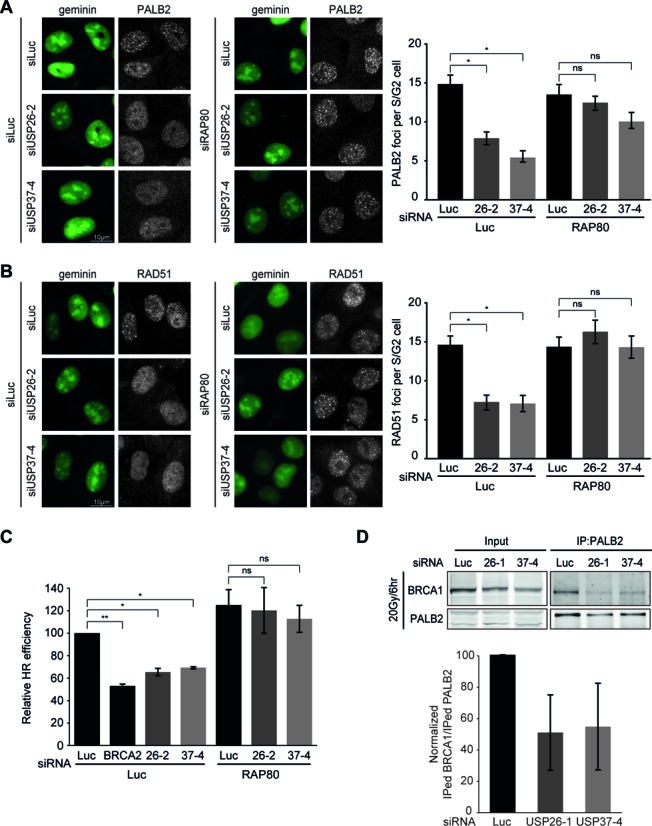
HR defects caused by the loss of USP26 and USP37 are reversed upon concomitant RAP80 removal. (**A**) Effect of DUB and RAP80 depletion on PALB2 (white) foci formation in mAG-geminin expressing (green) S/G2 cells. (**B**) As in A, but stained for RAD51 (white). (**C**) Effect of the indicated siRNAs on HR efficiency measured using the DR-GFP reporter. (**D**) Impact of DUB depletion on the interaction between PALB2 and BRCA1 as measured after immunoprecipitation (IP) of endogenous PALB2 6 h after 20 Gy of IR. Blots were probed for endogenous PALB2 and BRCA1. A representative western blot is presented. The graph shows the quantification of the immunoprecipitated amount of BRCA1 per amount of immunoprecipitated PALB2 from two independent experiments is. ns = non-significant, *P < 0.05, **P < 0.01, ***P < 0.001 (student's *t*-test). Quantified data are represented as mean ± S.E.M. (*n* = 2), except in (D) where mean ± S.D (*n* = 2) is shown.

To test the functional relevance of these findings, we depleted RAP80 and examined if this would restore HR proficiency in USP26 or USP37-depleted cells. Indeed, we found that the defective IR-induced accrual of both PALB2 and RAD51 in USP26- or USP37-depleted cells could be fully rescued by the additional depletion of RAP80 (Figure 6A and B). Similarly, HR efficiency was completely restored upon co-depletion of either DUB and RAP80, as measured in the DR-GFP reporter assay (Figure 6C). Cell-cycle profiles in these cells remained unchanged ruling out effects of cell cycle misregulation (Supplementary Figure S4F).

**Figure 7.**
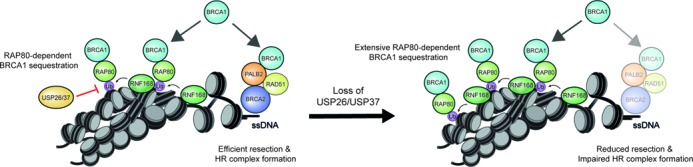
Molecular model for the role of USP26 and USP37 in HR. RNF168 sequesters BRCA1 away from HR sites through RAP80, which is functionally antagonized by USP26- and USP37-dependent de-ubiquitylation of RNF168-modified chromatin (see ‘Discussion’ section for details). Loss of USP26 or USP37 leads to more extensive RNF168-dependent sequestration of BRCA1, thereby preventing BRCA1 to complex and function with PALB2-BRCA2-RAD51 in HR. Additionally, the more extensive spreading of RAP80 reduces DNA end-resection, which also has a negative impact on HR.

One possible explanation for the HR defect in USP26- or USP37-depleted cells is that RAP80 promotes the extensive spreading of the HR-antagonizing BRCA1-A complex (38,40,54), thereby possibly affecting the formation of the HR-promoting BRCC complex (31,33). Pull-down of endogenous PALB2 or RAP80 showed that both these proteins interacted with BRCA1 as previously reported (34). However, we failed to detect interactions between PALB2 and RAP80 themselves, suggesting that BRCC and BRCA1-A are biochemically distinct BRCA1-containing complexes (Supplementary Figure S4G). Importantly, depletion of USP26 or USP37 markedly reduced the interaction between endogenous PALB2 and BRCA1, suggesting that the formation of BRCC complexes is impaired under conditions of extensive BRCA1-A spreading (Figure 6D). Together, these results suggest that USP26 and USP37 promote the BRCA1-dependent loading of PALB2 and RAD51 by counteracting the repressive impact of RAP80-dependent BRCA1 sequestration and RAP80-dependent inhibition of end-resection during HR (Figure 7).

## DISCUSSION

DSBs elicit a signaling cascade that is driven by the ubiquitin E3 ligases RNF8 and RNF168. These ligases promote progressive chromatin ubiquitylation, eventually culminating in the ubiquitin-dependent assembly of BRCA1, RAD18 and 53BP1 onto damaged chromosomes (7–9,13–16). However, while a clear picture of the factors that orchestrate the RNF8/RNF168 signaling pathway is emerging, we are only starting to understand how it is linked to DSBs repair (12,30,38,40,55–59).

In this study, we identify USP26 and USP37 as novel DUBs that reverse RNF168-mediated ubiquitylation (Figures 1 and 2, Supplementary Figures S1 and S2), a process known to repress HR by inhibiting DNA end-resection and possibly by sequestering the BRCA1-A complex through its ubiquitin-binding subunit RAP80 (38,40). By removing RNF168-induced ubiquitin conjugates distal from DSBs, USP26 and USP37 prevent the RAP80-dependent assembly of this BRCA1-containing complex, allowing BRCA1 to function in the BRCC complex during HR (Figure 7). These findings advance our conceptual understanding of the RNF168-dependent response to DSBs, by revealing pathways that orchestrate the spatial assembly and functioning of HR complexes at DSBs.

We propose the following model for RNF168-dependent regulation of HR (Figure 7): RNF168-induced ubiquitin conjugates spread away from DSBs into more distal chromatin regions (20). The BRCA1-A complex through RAP80 interactions associates with the RNF168-induced ubiquitin conjugates in these regions, thereby sequestering BRCA1 from the ssDNA compartment and inhibiting HR (38,40). At the same time, the recruitment of the BRCA1-A complex through RNF8/RNF168-induced ubiquitylation inhibits DNA end-resection and consequently HR (38,40). In line with this model, we demonstrate that supra-physiological levels of RNF168 triggered extensive ubiquitylation of H2A (Figure 2D), concomitant with a substantial reduction in HR efficiency (Figure 4B). We extend these findings by showing that this phenomenon is actively antagonized by USP26 and USP37. Loss of USP26/USP37 function markedly impairs the assembly of PALB2, RAD51 and efficient HR (Figures 3C and 6A–C). However, these defects can be rescued by the additional loss of RAP80 (Figure 6A–C). Mechanistically, we show that loss of USP26/USP37 causes extensive spreading of RNF8/RNF168-induced ubiquitin conjugates and ubiquitin-binding factors, such as RAP80 and BRCA1, into DSB-neighboring chromatin, which could be rescued by the additional loss of RAP80 (Figure 5A, B, D and Supplementary Figure S4E). These data suggest that USP26 and USP37 limit the magnitude of BRCA1-A complex assembly in DSB-neighboring chromatin by counteracting RNF168-mediated H2A ubiquitylation.

How does more extensive spreading of this complex inhibit HR? One possibility is that increased spreading of the BRCA1-complex limits the availability of BRCA1-A, thereby reducing the formation of the HR-promoting BRCC complex. In agreement with this hypothesis, we found that USP26 or USP37 depletion inhibits the efficient association of BRCA1 with PALB2, a unique subunit of the BRCC complex (Figure 6D). Together, this model may explain how USP26 and USP37 limit the repressive impact of RAP80 on HR.

An alternative, yet not mutually exclusive scenario, would be in line with recent findings showing that DSB-induced H2A/H2AX ubiquitylation needs to be reversed in the core of IRIF for DNA end-resection to occur (39). In accordance with this scenario, loss of USP26 or USP37 did indeed inhibit DNA end-resection (Figure 3D). Given that USP26 and USP37 are able to reverse RNF8/168-mediated ubiquitylation and promote HR, these enzymes would be ideal candidates to facilitate the repositioning of ubiquitylated substrates away from the DSB. However, the impact of USP26 and USP37 on the recruitment of PALB2 and RAD51 was much more severe than its impact on DNA end-resection. A similar phenotype was also observed in cells depleted for BRCA1 (60). We therefore favor a scenario in which these DUBs primarily regulate HR at the level of BRCA1. In addition to HR, we also found USP26 or USP37 to promote efficient DSB repair by NHEJ. Although the mechanism underlying the regulation of NHEJ by these DUBs is currently unclear, it is possible that these enzymes may act on ubiquitylated repair factors, such as KU80 and 53BP1, especially considering that RNF8 and RNF168 have been identified as the E3 ubiquitin ligase targeting these NHEJ-related factors (27,61).

Several other DUBs that affect H2A ubiquitylation have been identified as important players in the DDR. For instance, the activity of tumor suppressor BAP1, which de-ubiquitylates H2A at K119, appeared to be critical for efficient HR (50,62). However, whether the BAP1-dependent removal of this histone modification is important during HR remains unclear. Similar to USP26 and USP37, two other DUBs, USP3 and USP44, were shown to reverse RNF168-induced chromatin ubiquitylation, thereby controlling the accumulation of BRCA1 and 53BP1 at sites of DNA damage (21,26,63). Future work will reveal whether these DUBs, similarly to USP26 and USP37, operate to control DSB repair, in particular HR. Additionally, another DUB, OTUB2, was recently found to regulate DNA-end resection and therefore DSB repair pathway choice (25). Unraveling the interplay between different DUBs during HR may uncover how ubiquitin-dependent control of this important DNA damage repair process is orchestrated.

Notably, both USP26 and USP29 are retrogenes of USP37, which likely explains why these DUBs display certain functional similarities. Although USP26 is often considered as testis-specific, we were able to detect USP26 expression in different cell types (U2OS and HEK293, Supplementary Figure S3A and B), showing that this classification is incorrect. In agreement, extensive proteomic analysis has revealed that USP26 is expressed in various human cell-lines and organs (64,65). Moreover, knockdown of USP26, similar to that of USP37, confers defects in the signaling and repair of DSBs in human cells, suggesting non-redundant roles for both DUBs in the DSB response. Indeed, co-depletion of these DUBs did not aggravate defects in the DSB response further than the depletion of either DUB alone (Supplementary Figure S3D and F).

A striking conclusion from our study is that both over-expression as well as knockdown of USP26 or USP37 impairs HR. We report here that these DUBs limit the repressive impact of RAP80 on HR explaining how knockdown of these DUBs affects HR. Clearly, the fact that over-expression and knockdown cause the same phenotype indicates that these DUBs can regulate HR through distinct mechanisms. Future studies on these mechanisms may be crucial to further uncover how these DUBs regulate HR. Considering that non-physiological expression of these DUBs impairs HR it seems likely that the expression of USP26 and USP37 needs to be tightly controlled. Failure to do so might not only correlate with enhanced genomic instability, but also with increased malignant transformation rates. Indeed, a plethora of cancer cell lines appear to have either lost (USP26 = 1457/USP37 = 446; COSMIC) or amplified (USP26 = 309/USP37 = 359; COSMIC) the expression of these DUBs. In conclusion, we report distinct ubiquitin-dependent pathways that orchestrate the assembly and function of HR complexes at DSBs and identify the factors responsible for these events.

## SUPPLEMENTARY DATA

Supplementary Data are available at NAR Online.

SUPPLEMENTARY DATA
