# Supplementary material for: The de-ubiquitylating enzymes USP26 and USP37 regulate homologous recombination by counteracting RAP80
Source: Nucleic Acids Res. 2015 Jun 22;43(14):6919–33. doi: 10.1093/nar/gkv613 (PMC4538816; doi:10.1093/nar/gkv613)
Supplement: SUPPLEMENTARY DATA [file supp_43_14_6919__index.html]

The de-ubiquitylating enzymes USP26 and USP37 regulate homologous recombination by counteracting RAP80 — The de-ubiquitylating enzymes USP26 and USP37 regulate homologous recombination by counteracting RAP80 — SUPPLEMENTARY DATA 

# The de-ubiquitylating enzymes USP26 and USP37 regulate homologous recombination by counteracting RAP80

## SUPPLEMENTARY DATA

- SUPPLEMENTARY DATA
