# Supplementary material for: The de-ubiquitylating enzymes USP26 and USP37 regulate homologous recombination by counteracting RAP80
Source: Nucleic Acids Res. 2015 Jun 22;43(14):6919–33. doi: 10.1093/nar/gkv613 (PMC4538816; doi:10.1093/nar/gkv613)
Supplement: SUPPLEMENTARY DATA [file supp_gkv613_nar-00131-d-2015-File009.pdf]

Supplemental Figure Legends

Supplemental Figure 1. A DUB over-expression screen reveals novel DSB response regulators. (A) List of the FLAG-tagged DUBs expressed in U2OS cells. Enzymes are color-coded to signify the different DUB families. In yellow Ubiquitin C-terminal Hydrolases (UCH), in green Machado-Josephin domain-containing proteins (MJD), in blue JAMM metalloproteases (JAMM), in red Otubain domain-containing proteins (OTU), in purple unclassified DUBs and in grey Ubiquitin Specific Proteases (USP). (B) Quantification of 53BP1 IRIF formation upon over-expression of the indicated DUBs. Red bars signify hits, i.e. population of cells with >5 53BP1 IRIFs is lower than 15% of total transfected cells. (C) Quantification of RAD51 IRIF formation upon over-expression of the indicated DUBs. Red bars signify hits, i.e. population of cells with >10 RAD51 IRIFs is lower than 15% of total transfected cells. (D) Examples of the impact of the over-expression of the indicated (non-) hits/DUBs (green) on 53BP1 IRIF formation (white). (E) Examples of the impact of the over-expression of the indicated (non-) hits/DUBs (green) on RAD51 IRIF formation (white).

Supplemental Figure 2. USP26 and USP37 accumulate at DSBs and regulate chromatin ubiquitylation. (A) Quantification of the impact of the expression of GFP- or mCherry-tagged DUBs (green or red) on γH2AX, MDC1, FLAG-RNF8 IRIF formation. (B) As in A, except that RNF168, FK2, RAP80 and BRCA1 IRIF formation was quantified. In addition, the effect of the expression of catalytically inactive mCherry-tagged DUBs on FK2 IRIF was determined. (C) Recruitment of mCherry-tagged DUBs (red) to γH2AX-

marked DNA damage sites (green) after UV-A laser micro-irradiation. (D) Quantification of the effect of the expression of GFP-tagged DUBs (green) on the formation of ubiquitylated substrates (FK2; white) upon chromatin tethering of mCherry-RNF168 (red). (E) As in D, but for 53BP1 (white). (F) Western blot analysis of ubiquitylation levels upon expression of the indicated mCherry-tagged DUBs. Blots were probed for unmodified and ubiquitylated H2B, as well as for mCherry and K48-, K63- and total (FK2)-ubiquitin. ns=non-significant, *, P<0.05, **, P<0.01, ***, P<0.001 (student's t test). Quantified data are represented as mean ± S.D. (n=3).

Supplemental Figure 3. USP26 and USP37 have non-redundant roles in the DSB response. (A) Relative USP26 mRNA expression in U2OS cells treated with the indicated siRNAs. The USP26 transcript was not detectable (ND) by reverse transcriptase (RT)-qPCR in cells treated with siRNAs against USP26 (left panel). Representative agarose gel showing USP26 PCR product amplified from total RNA without (-RT) or with (+RT) reverse transcriptase reaction (right panel). (B) Relative USP37 mRNA expression in U2OS cells treated with the indicated siRNAs (lower panel). Relative endogenous USP37 protein expression in VH10-SV40 immortalized fibroblasts or U2OS cells treated with the indicated siRNAs. Tubulin is a loading control (upper panels). (C) Effect of DUB depletion on 53BP1 IRIF formation in time after 2 Gy of IR. (D) Effect of single- or double-DUB depletion on 53BP1 IRIF after 2 Gy of IR. (E) FACS plot of the engineered U2OS cells stably expressing mAG-geminin. The y-axis represents mAG (green) fluorescence intensity, while the x-axis shows DNA content. Different cell-cycle stages are indicated by vertical lines.

(F) Impact of single- or double-DUB depletion on HR efficiency measured using the DR-GFP reporter. ns=non-significant, *, $P<0.05$, **, $P<0.01$, ***, $P<0.001$ (student's t test). Quantified data are represented as mean ± S.D. (n=3).

Supplemental Figure 4. Loss of USP26 or USP37 affects the DSB response, but not cell cycle progression. (A) Cell cycle profiles of cells treated with the indicated siRNAs. (B) Cell cycle profiles of cells over-expressing the indicated GFP fusion proteins. (C) Effect of combined DUB depletion on DSB repair, assayed by clearance of γH2AX foci after 2 Gy of IR. (D) Effect of DUB and RAP80 depletion on RAP80 IRIF formation. (E) Impact of DUB depletion on the relative position/expansion of the indicated factors (FK2 or 53BP1; green) upon DSB-induction by FokI-mCherry-LacR (red). (F) Cell cycle profiles of cells treated with the indicated siRNAs. (G) BRCA1-PALB2-BRCA2-RAD51- (BRCC) and BRCA1-Abraxas-RAP80-MERIT40- (BRCA1-A) complexes are distinct, as observed upon immunoprecipitation (IP) of endogenous PALB2 or RAP80. Blots were probed for endogenous BRCA1, PALB2, RAP80, BRCC36, RAD51 and H2AX. ns=non-significant, *, $P<0.05$, **, $P<0.01$, ***, $P<0.001$ (student's t test). Quantified data are represented as mean ± S.D. (n=3), except in (D) and (E) where mean ± S.E.M. (n=2) is shown.

Typas_Suppl_Fig.1

A

UCH	MJD	JAMM	OTU	Unclassified	USP				
UCHL1	JOSD1	STAMBP	OTUB1	PARP11	USP1	USP2	USP30	USP43	USP52
UCHL3	JOSD2	STAMBPL1	OTUB2	PSMD7	USP10	USP21	USP33	USP44	USP53
BAP1	JOSD3	BRCC36	OTUD4	PSMD14	USP11	USP22	USP36	USP45	USP7
	ATXN3	EIF3S3	OTUD5	VCPIP1	USP13	USP25	USP37	USP46	USP8
		EIF3S5	OTUD6B		USP14	USP26	USP38	USP48	USPL1
		COPS5	OTUD7B		USP15	USP28	USP39	USP49	DUB3
		COPS6			USP18	USP29	USP4	USP5	CYLD
					USP19	USP3	USP42	USP50	

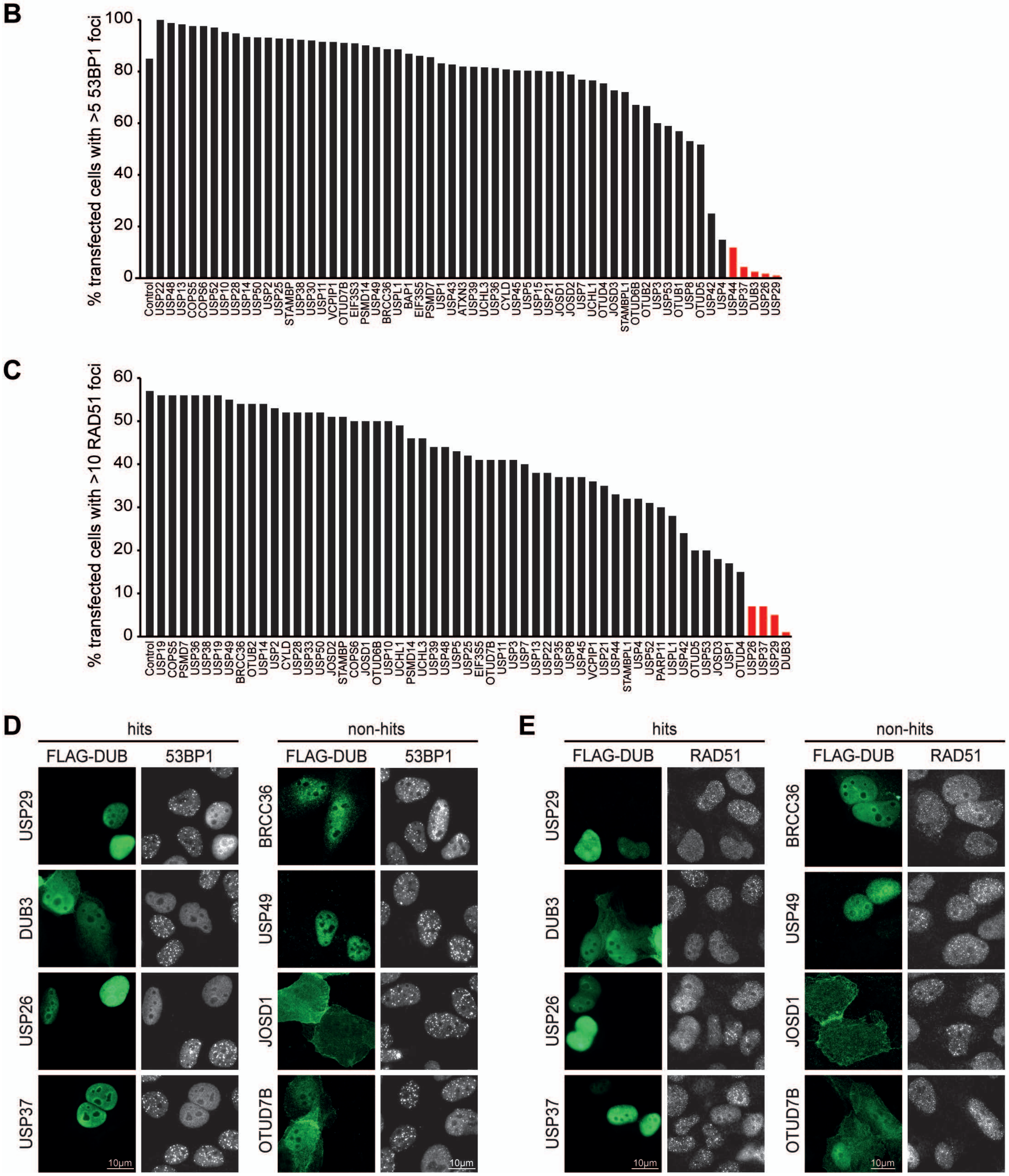

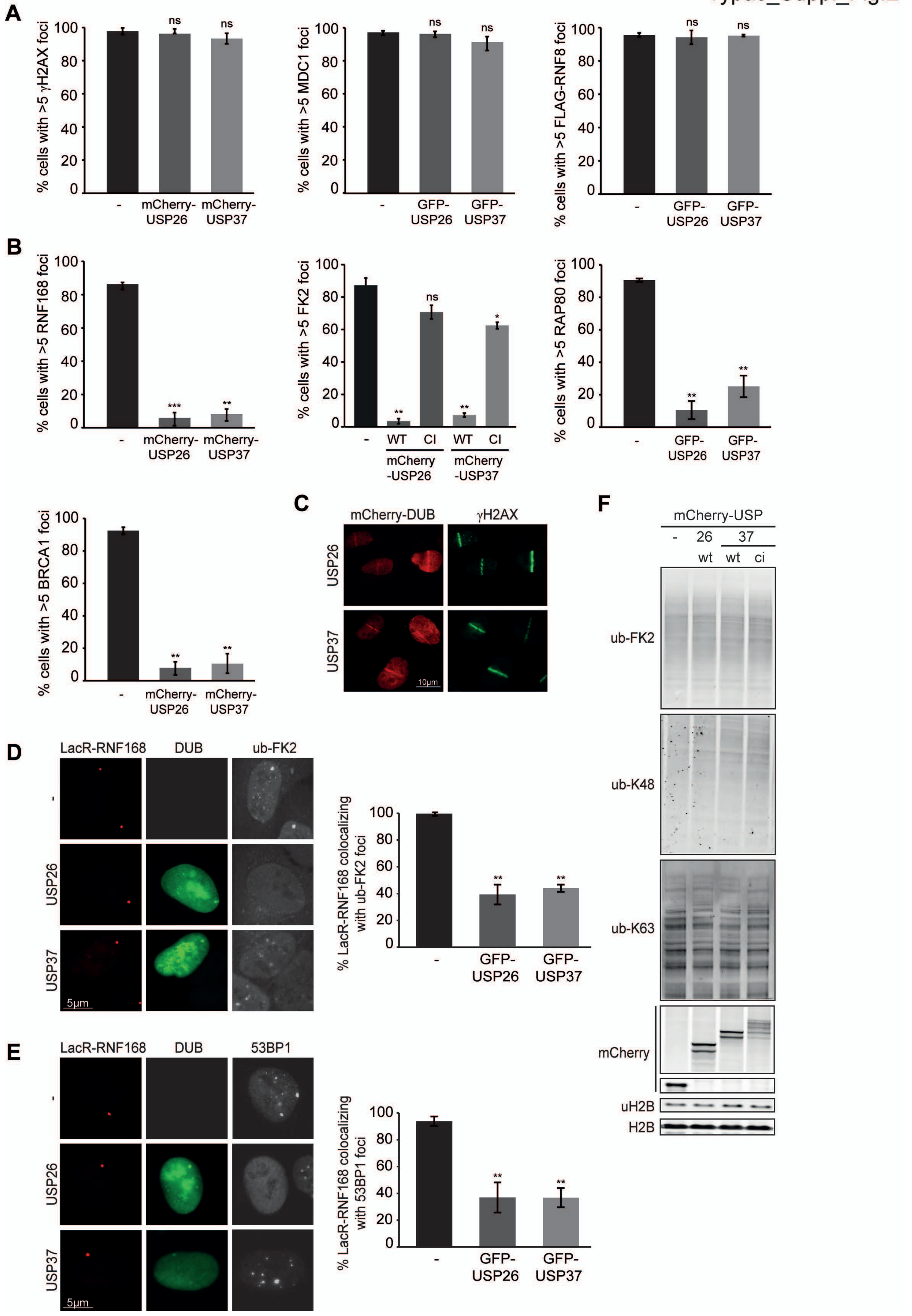
A
% cells with >5 γH2AX foci
mCherry-USP26
mCherry-USP37
ns
% cells with >5 MDC1 foci
GFP-USP26
GFP-USP37
% cells with >5 FLAG-RNF8 foci
B
% cells with >5 RNF168 foci
% cells with >5 FK2 foci
WT
CI
mCherry-USP26
mCherry-USP37
% cells with >5 RAP80 foci
% cells with >5 BRCA1 foci
C
mCherry-DUB
γH2AX
USP26
USP37
10µm
D
LacR-RNF168
DUB
ub-FK2
5µm
% LacR-RNF168 colocalizing with ub-FK2 foci
E
53BP1
% LacR-RNF168 colocalizing with 53BP1 foci
F
mCherry-USP
26
37
wt
ci
ub-FK2
ub-K48
ub-K63
mCherry
uH2B
H2B

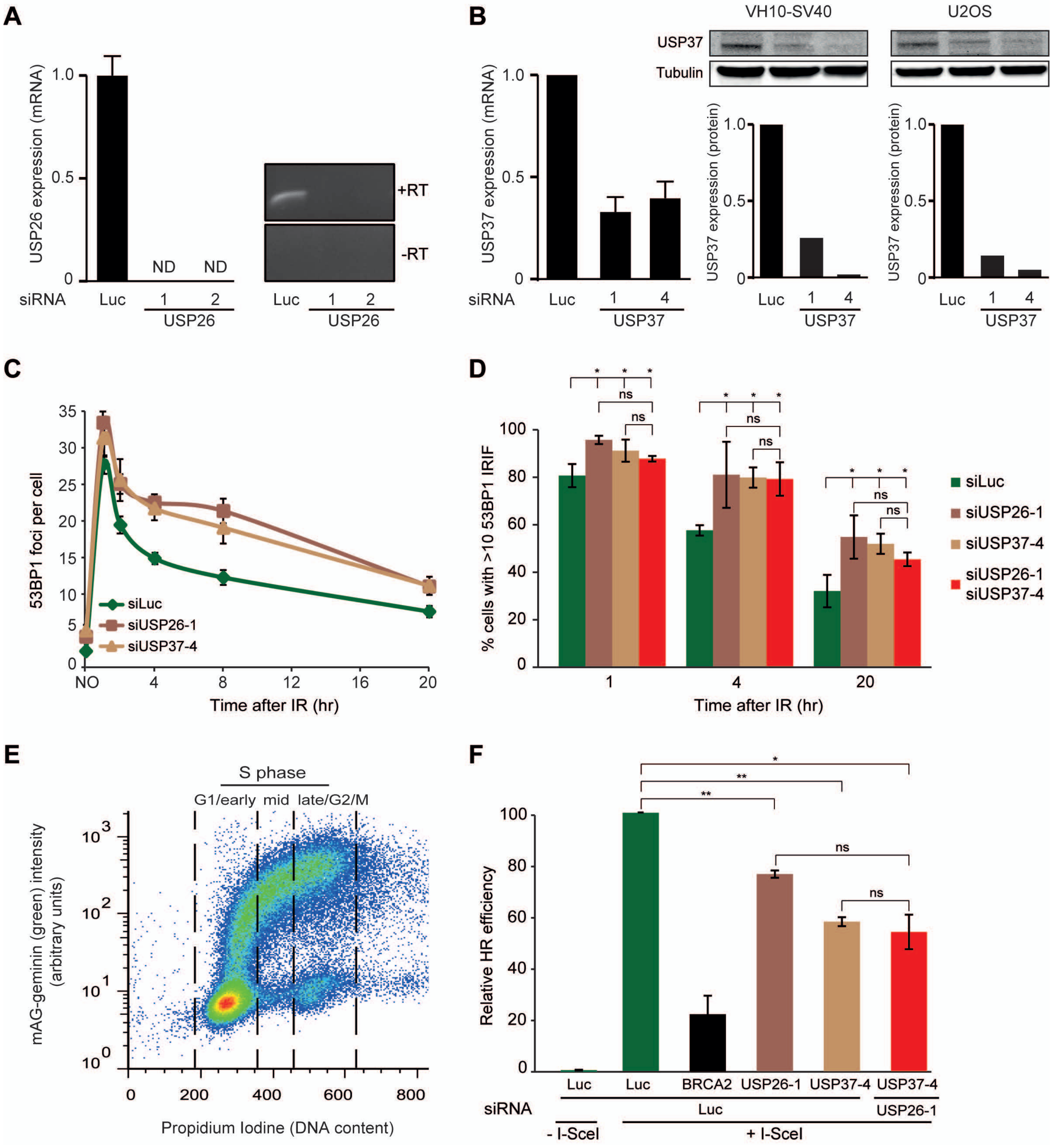
A
USP26 expression (mRNA)
1.0
0.5
0
ND
ND
siRNA
Luc
1
2
USP26
+RT
-RT
Luc
1
2
USP26
B
VH10-SV40
U2OS
USP37
Tubulin
USP37 expression (mRNA)
siRNA
Luc
1
4
USP37
USP37 expression (protein)
C
53BP1 foci per cell
siLuc
siUSP26-1
siUSP37-4
NO
Time after IR (hr)
D
% cells with >10 53BP1 IRIF
ns
siLuc
siUSP26-1
siUSP37-4
siUSP26-1
siUSP37-4
Time after IR (hr)
E
S phase
G1/early mid late/G2/M
mAG-geminin (green) intensity
(arbitrary units)
Propidium Iodine (DNA content)
F
Relative HR efficiency
Luc
Luc
BRCA2
USP26-1
USP37-4
USP37-4
siRNA
Luc
USP26-1
- I-SceI
+ I-SceI

Typas_Suppl_Fig.4

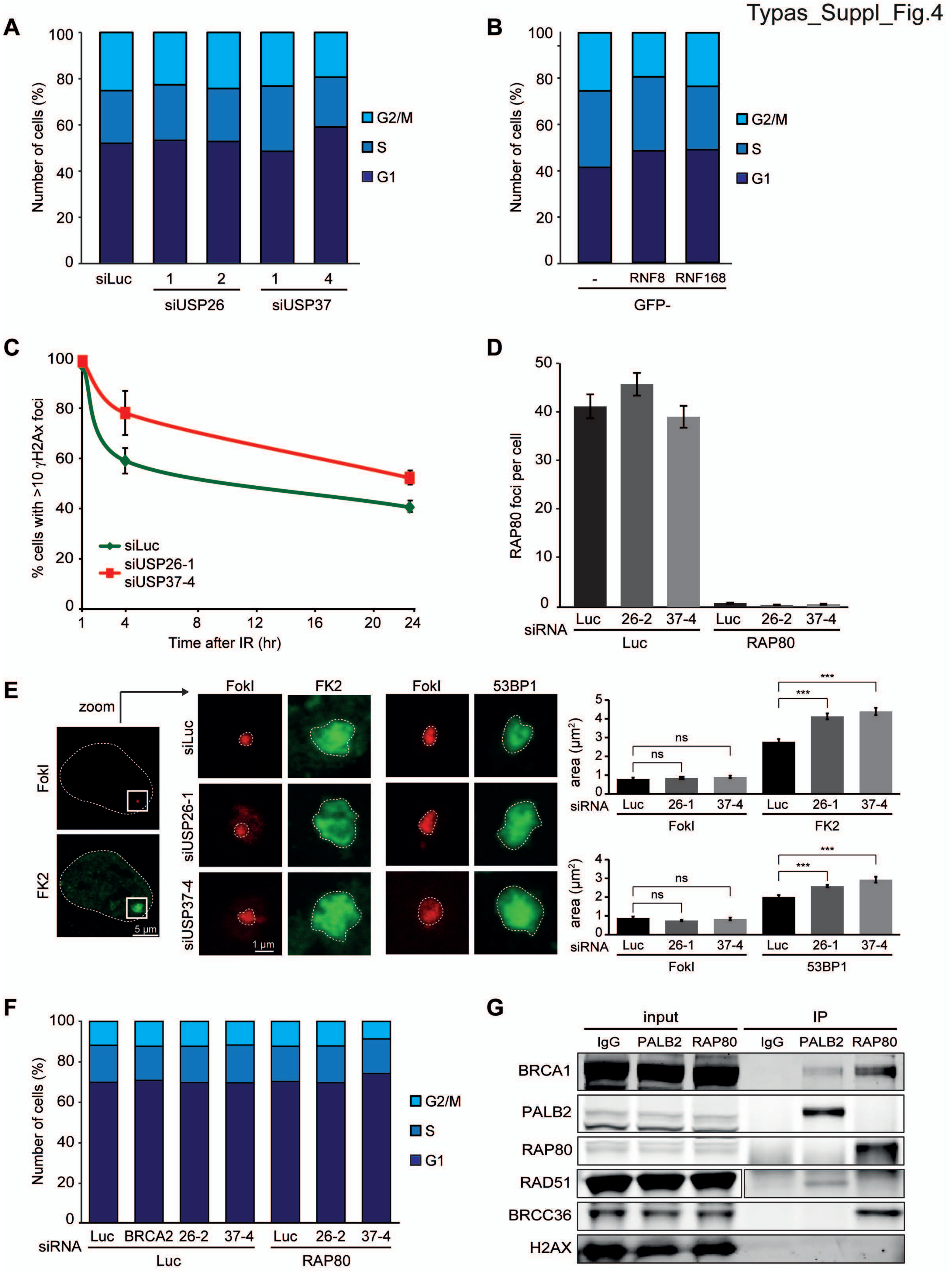

Supplemental Table

Antibodies					
**Target protein**	**Host**	**Obtained from**	**Cat. nr.**	**IF**	**WB**
53BP1	rabbit	Novus Biologicals	NB100-304	1:2000	
BRCA1	mouse	SantaCruz	sc-6954	1:100	
BRCA1	rabbit	gift Dr. Daniel Durocher			1:1000
BRCC36	rabbit	Abcam	ab62075		1:1000
FLAG	mouse	Sigma	F1804	1:1000	
GFP	rabbit	Abcam	ab290		1:1000
GFP	mouse	Roche	#11814460001		1:2000
γH2AX	mouse	Millipore	JBW301	1:2000	
H2AX	rabbit	Bethyl	A330-082A		1:10000
H2B	rabbit	Millipore	07-371		1:10000
HA	mouse	SantaCruz	sc-7392	1:500	
mCherry	mouse	Abcam	ab125096		1:1000
MDC1	rabbit	Abcam	ab11171	1:1000	
PALB2	rabbit	Bethyl	A301-246A		1:1000
PALB2	rabbit	gift Dr.Bing Xia		1:100	
RAD51	rabbit	SantaCruz	sc-8349	1:100	
RNF168	rabbit	Millipore	ABE367	1:200	1:500
Tubulin	mouse	Sigma	T6199		1:5000
Ubiquitin K48 chains	rabbit	Millipore	APU2/05-1307		1:100
Ubiquitin K63 chains	rabbit	Millipore	APU3/05-1308		1:1000
Ubiquitin-FK2	mouse	EnzoLifeSciences	BML-PW8810-0500	1:100	
RPA	mouse	ThermoScientific	Ab-1 9H8	1:1000	
CtIP	mouse	gift Dr. Richard Baer		1:10	
RAP80	rabbit	Bethyl	A300-764		1:1000
RAP80	rabbit	Bethyl	A300-763A	1:500	
Rabbit-700CW	donkey	Licor			1:20000
Mouse-800CW	donkey	Licor			1:20000

siRNAs		
**Target Protein**	**siRNA name**	**Sequence (5'-3')**
Luciferase	Luc	CGUACGCGGAAUACUUCGA
BRCA2	BRCA2	GAAGAAUGCAGGUUUAAUA
RNF8	RNF8-1	GAGGGCCAAUGGACAAUUA
USP26	USP26-1	CCACAAAGCUGGAGGUAAA
USP26	USP26-2	CCACAAAGUUGAUGAGAAA
USP26	USP26-3	CAGAAGAGCTTGAGTATAA
USP37	USP37-1	CUACAAUACUGGAGGAAUU
USP37	USP37-4	GAAGAUUACCCUAAGGAAA
RAP80	RAP80<br>(Dharmacon<br>SMARTpool<br>L-006995-00-0005)	GTAAATCCCTGGTCCCATT<br>AAATGAATCTCCCGTCAAG<br>AGAGCAGGCTAGTGAGAAA<br>AGAGGCAGCTCCTTAATAA

qPCR primers		
**Target gene**	**Gene/Primer**	**Sequence (5'-3')**
USP26	USP26-Forward	AGTGTTGTCAGCCATCTTGG
USP26	USP26-Reverse	CCCGCATATCATCGTAAGTG
USP37	USP37-Forward	GCCCAAAACAATCACAGAGC
USP37	USP37-Reverse	TCCCTTTCACGCTCCATATC

Plasmids	
**Encoded fusion protein**	**Described in**
GFP-H2A	Luijsterburg *et al. Journal of Cell Biology* **197**: 267-281 (2012)
FokI-LacR-mCherry	Tang *et al. Nature Structural & Molecular Biology* **20**: 317-325 (2013)
mCherry-LacR-RNF168	Luijsterburg *et al. EMBO Journal* **31**: 2511-2527(2012)
mCherry-LacR-RNF8	Luijsterburg *et al. EMBO Journal* **31**: 2511-2527(2012)
mCherry-RNF168	Smeenk *et al. Journal of Cell Science* **126**: 889-903 (2013)
mCherry-RNF8	Smeenk *et al. Journal of Cell Science* **126**: 889-903 (2013)
